# Construction of a neural network diagnostic model and investigation of immune infiltration characteristics for Crohn’s disease

**DOI:** 10.3389/fgene.2022.976578

**Published:** 2022-09-15

**Authors:** Yufei Yang, Lijun Xu, Yuqi Qiao, Tianrong Wang, Qing Zheng

**Affiliations:** Division of Gastroenterology and Hepatology, Key Laboratory of Gastroenterology and Hepatology, Ministry of Health, Inflammatory Bowel Disease Research Center, Ren ji Hospital, School of Medicine, Shanghai Jiao Tong University, Shanghai Institute of Digestive Disease, Shanghai, China

**Keywords:** inflammatory bowel disease, Crohn’s disease, immune cells, bioinformatics, artificial neural network model

## Abstract

**Objective:** Crohn’s disease (CD), a chronic recurrent illness, is a type of inflammatory bowel disease whose incidence and prevalence rates are gradually increasing. However, there is no universally accepted criterion for CD diagnosis. The aim of this study was to create a diagnostic prediction model for CD and identify immune cell infiltration features in CD.

**Methods:** In this study, gene expression microarray datasets were obtained from the Gene Expression Omnibus (GEO) database. Then, we identified differentially expressed genes (DEGs) between 178 CD and 38 control cases. Enrichment analysis of DEGs was also performed to explore the biological role of DEGs. Moreover, the “randomForest” package was applied to select core genes that were used to create a neural network model. Finally, in the training cohort, we used CIBERSORT to evaluate the immune landscape between the CD and normal groups.

**Results:** The results of enrichment analysis revealed that these DEGs may be involved in biological processes associated with immunity and inflammatory responses. Moreover, the top 3 hub genes in the protein-protein interaction network were IL-1β, CCL2, and CXCR2. The diagnostic model allowed significant discrimination with an area under the ROC curve of 0.984 [95% confidence interval: 0.971–0.993]. A validation cohort (GSE36807) was utilized to ensure the reliability and applicability of the model. In addition, the immune infiltration analysis indicated nine different immune cell types were significantly different between the CD and healthy control groups.

**Conclusion:** In summary, this study offers a novel insight into the diagnosis of CD and provides potential biomarkers for the precise treatment of CD.

## Introduction

Inflammatory bowel disease (IBD) includes two main types, ulcerative colitis and Crohn’s disease (CD) (Zhang and Li, 2014). The area of damage in CD is the entire gastrointestinal tract and extra-intestinal sites. In the absence of a gold standard for diagnosis, we usually diagnose patients through comprehensive considerations based on clinical features, endoscopy, and imaging examinations (Wehkamp et al., 2016). In most cases, it is difficult to diagnose CD and distinguish when patients are in the early stages of disease. However, it is widely recognized that an early diagnosis and treatment have a better prognosis than CD with complications such as intestinal obstruction, anal fistula, and perianal abscess. Therefore, it is necessary to explore potential biomarkers to provide a precise diagnosis and effective therapy.

Although little is known about its pathogenesis, there is convincing evidence that genetic susceptibility, immune imbalance, change in environmental factors, and intestinal microbiota disorders are contributors to CD pathogenesis (Seyedian et al., 2019). It is now obvious that CD is an autoimmune disease characterized by an imbalance of the immune system (Li and Shi, 2018). Therefore, the role of immune cells in CD pathogenesis needs to be investigated further.

Recently, the development of high-throughput sequencing technology has laid the foundation for precision medicine. Many machine learning algorithms, such as random forest and support vector machine, have been developed and used in various research fields (Bao et al., 2022). Bao et al. proposed a new identification algorithm to accurately identify modified residues, which has contributed to bioinformatics and research related to diseases and the development of drugs (Bao et al., 2021). However, many investigators require an operational model for the early identification and diagnosis of CD. Recently, Chen et al. developed and validated a predictive nomogram for CD, which can identify potential biomarkers for a diagnosis of CD (Chen et al., 2020). However, few studies have focused on the construction of a predictive model for CD diagnosis from the perspective of an artificial neural network (ANN).

A neural network combined with artificial intelligence has gradually been applied to the medical field to help physicians cope with large amounts of data and implement precision medicine more conveniently. ANN is a type of deep learning, which was inspired by the human brain. The specific algorithm of artificial neural networks is based on the learning and trail-and-error techniques. Previous studies of ANN mainly focused on the prediction and prognosis of tumors (Kourou et al., 2015; Daoud and Mayo, 2019; Albaradei et al., 2021). Our study aimed to establish an artificial neural network model for CD based on the weight of candidate genes and to investigate the different immune cell types between the CD and control groups. Given the superiority of this model in this study, it can be used to effectively distinguish between CD samples and normal samples, which will be of great significance for the diagnosis of CD in the future.

## Materials and methods

### Data collection and analysis

Three gene expression microarray datasets (GSE16879, GSE112366, and GSE36807) were selected from the Gene Expression Omnibus (GEO) database, an internationally available repository that provides several web-based tools to help users browse and download data. GSE16879 and GSE112366, whose platforms were based on GPL570 and GPL13158, respectively, were merged as a training cohort. The dataset GSE36807, also based on the platform GPL570, was used as a validation cohort. Three eligible datasets were incorporated into this study and the related information of the datasets is shown in Table 1.

**TABLE 1:** Baseline characteristics of the training and validation cohorts.

Dataset ID	Platform	Crohn’s disease	Normal	Total
GSE16879	GPL570	37	12	49
GSE112366	GPL13158	141	26	167
GSE36807	GPL570	13	7	20

### Screening of DEGs and enrichment analysis

We selected DEGs in the CD and control groups using the “limma” package in R software. A |log-fold change (FC)|>1.2 and adjusted *p-*values < 0.05 were considered significant criteria to screen DEGs. To visualize the results, R package “ggplot2” and “pheatmap” were applied to transform the outcome into a volcano plot and heat map. To investigate whether there was differential expression between different phenotypes in CD, we excluded healthy samples in the training set and divided the biopsy samples into two groups according to different disease locations: colon and ileum groups. We selected DEGs between the colon and ileum groups using the same method. The result is also presented in the heat map. GO and KEGG enrichment analyses were performed to explore the biological functions and pathways of the DEGs between the CD and healthy control groups. An adjusted *p*-value < 0.05 was considered the cutoff value. The results of GO and KEGG enrichment analysis are shown as a bubble plot.

### Protein-protein interaction (PPI) network construction and hub genes investigation

To study the functional connections of DEGs from a protein perspective, STRING analysis databases, common online platforms (https://cn.string-db.org/cgi/input.pl), include data on the physical interactions and full network function associations of proteins. Before constructing the PPI Network, we studied high-scoring physical interactions at the protein level. Only genes with a score >0.7 in STRING were considered significant and used to construct the PPI Network. The results were visualized by Cytoscape.

### Identifying candidate genes by random forest

The R package “randomForset” was utilized to develop a random forest model to further screen out DEGs. First, 500 trees were seen as a variable of the model and the average miscalculation of all variables in the two groups was calculated. The optimal tree number was obtained by calculating the lowest error rate of cross validation. Next, a random forest was created according to the calculated parameters. Finally, the top 30 DEGs were identified as candidate genes based on the decreasing accuracy method to construct the ANN.

### Construction and validation of the ANN prediction model

The first step for model construction was expression data processing, which transformed the 30 DEGs into a “Gene Score”. The score of candidate genes was analyzed based on their expression levels, which were compared with the median of all sample expression values. When the expression level of an upregulated gene is lower, it will receive a score of 0, otherwise it is assigned a value of 1. When the expression level of a downregulated gene is lower, it will receive a score of 1, otherwise it is assigned a value of 0.

Then, we developed a “Gene Score” sheet, which included 216 lines of samples and 30 columns of DEGs. The R package “neuralnet” was used to develop an ANN model, which included five hidden layers. In this model, the result of the output layer was determined by the sum of the gene score multiplied by the gene weight. Considering the potential impact of biopsy samples in different disease locations on the diagnostic model, we performed binary logistic regression to analyze the influence of disease location on the diagnosis of CD. A *p*-value < 0.05 was considered statistically significant. The performance of the model was assessed by ROC analysis and presented as the area under the ROC curve (AUC). To verify the applicability of the model, the microarray data of GSE36807 were obtained from the GEO database and the genetic expression information was transformed into a gene score in the same way.

### Immune infiltration analysis

CIBERSORT is an algorithm that accurately estimates the immune cell composition of tissues based on gene expression profiles. We obtained the proportion of 22 types of immune cells between CD and healthy individuals. A *p*-value <0.05 was considered the filtration condition and the findings were visualized in a bar plot. We used the R package “corrplot” to analyze the correlation of 22 types of immune cells, which was visualized in a heatmap. The R package “vioplot” was used to investigate the difference in infiltrating immune cells between CD and control samples.

## Results

### Identification of DEGs

**FIGURE 1:**
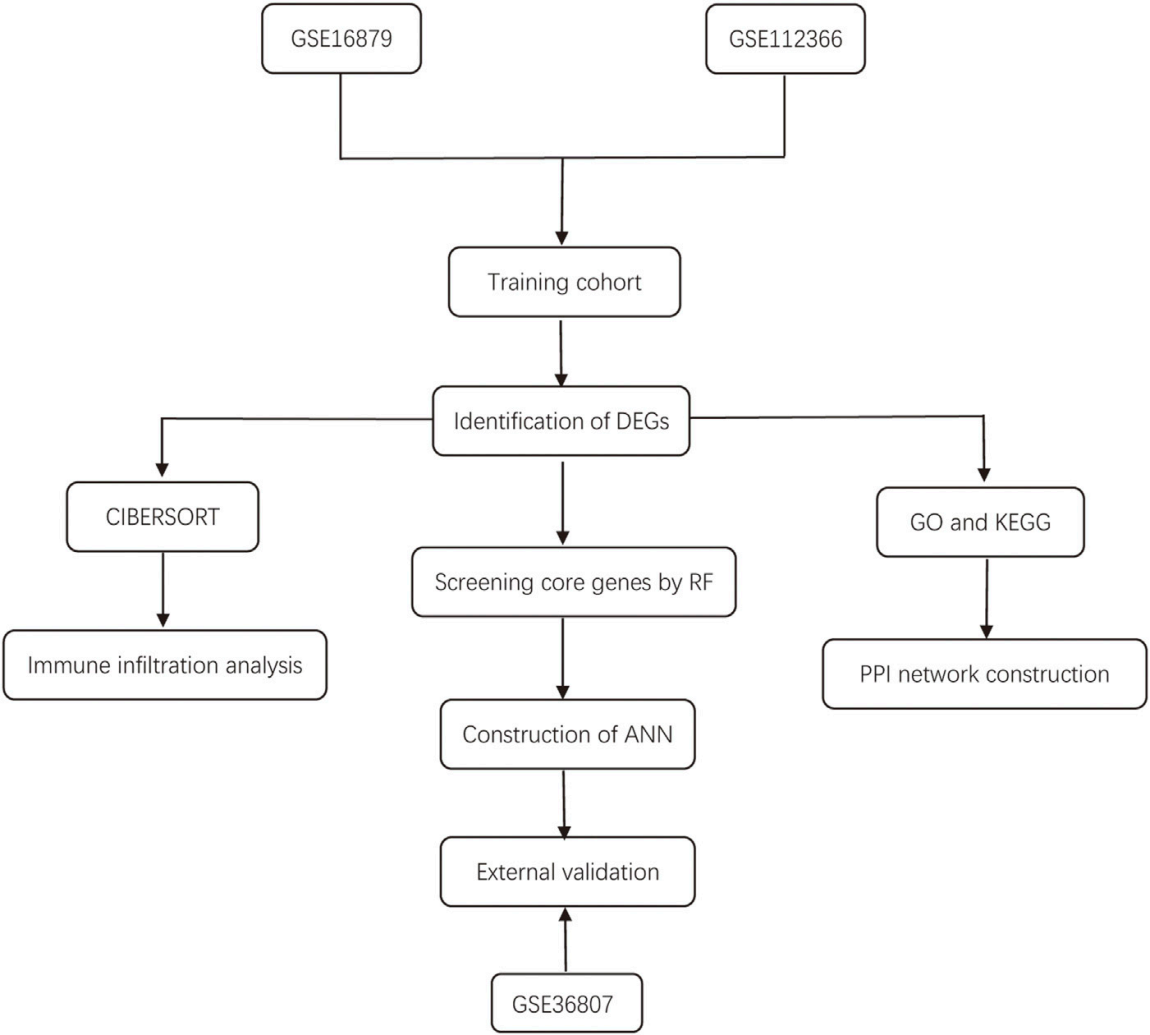
Flow diagram of the study design.

**FIGURE 2:**
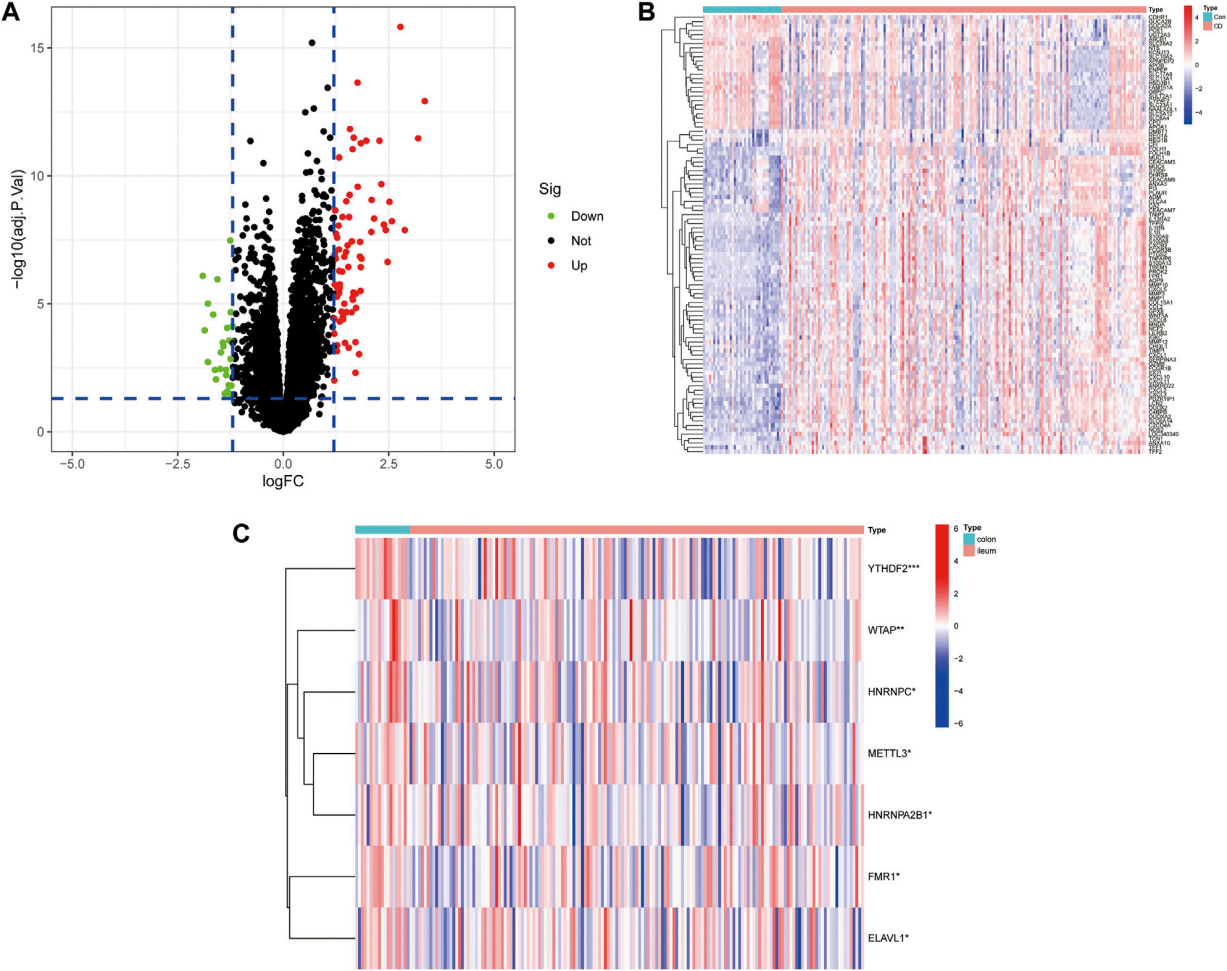
**(A)** A volcano plot of differentially expressed genes between Crohn’s disease and control groups. **(B)** Heatmap of DEGs between Crohn’s disease and control groups. **(C)** Heatmap of DEGs between colon and ileum groups in the Crohn’s disease group.

The workflow of the study is illustrated in Figure 1. In this study, we merged the datasets GSE16879 and GSE112366 into a training cohort and removed the batch effect using the “Sva” package. The training cohort included 216 samples, of which 178 were from CD biopsy samples and 38 were from healthy biopsy samples. According to the foregoing criteria, 102 DEGs were determined by using the “limma” package from 16,933 genes in the training set (Figure 2A, Figure 2B). DEGs of different disease phenotypes in CD is presented in Figure 2C.

### GO enrichment and KEGG pathway analysis of DEGs

**FIGURE 3:**
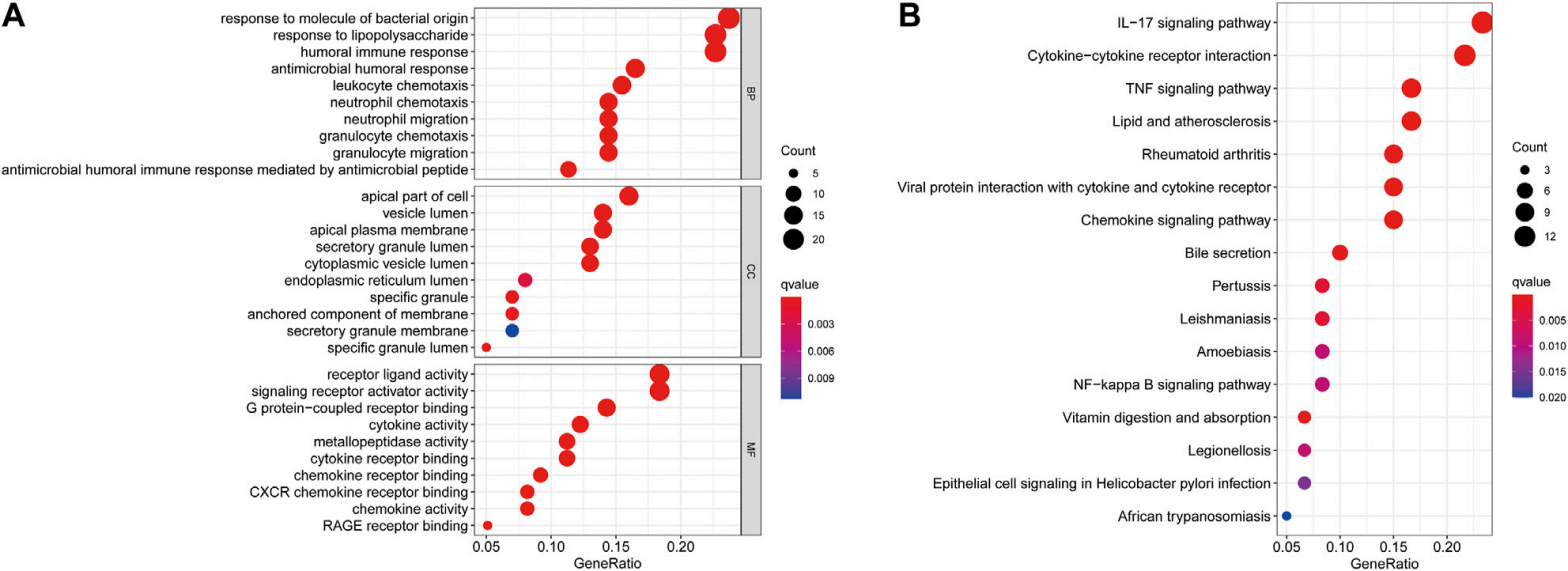
GO **(A)** and KEGG **(B)** enrichment analysis of differentially expressed genes between the CD and healthy control groups.

As shown in Figure 3A, GO enrichment analysis demonstrated that DEGs were involved in biological processes associated with immune responses and inflammatory responses, including humoral immune responses, responses to molecules of bacterial origin, neutrophil chemotaxis and migration, granulocyte chemotaxis and migration, cytokine and chemokine activity, and CXCR chemokine receptor binding. KEGG pathway analysis revealed that DEGs were mainly enriched in the inflammatory signaling pathway, including the IL-17 signaling pathway, TNF signaling pathway, and chemokine signaling pathway, which have a vital role in the development of CD (Figure 3B).

### PPI network analysis and selection of hub genes

**FIGURE 4:**
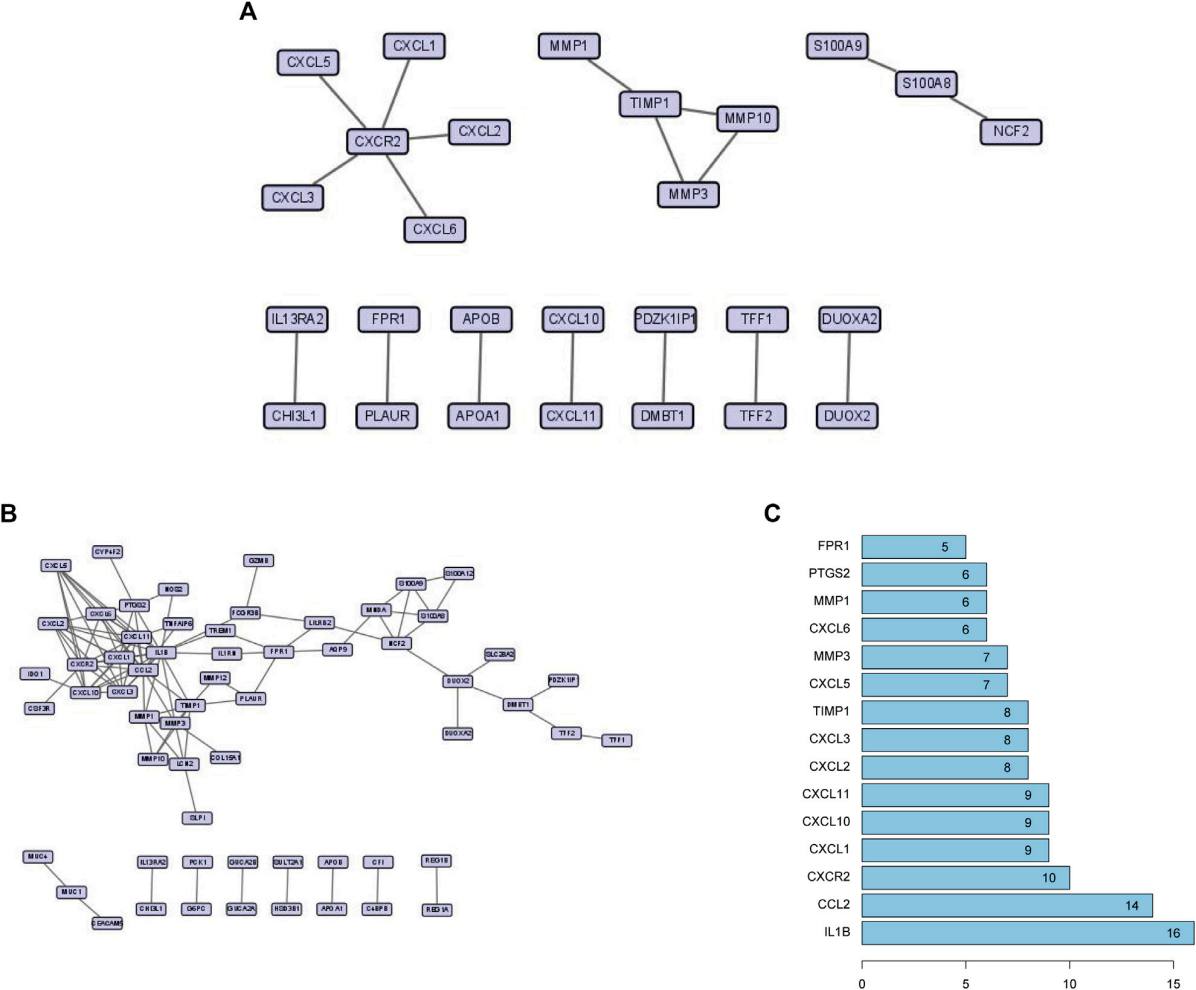
**(A)** High-scoring physical interactions from the STRING network. **(B)** A protein-protein interaction network of differentially expressed genes. **(C)** The top 15 hub genes and their degree values of modules.

The physical interaction of proteins in the STRING network is shown in Figure 4A. To explore the related information of DEGs at the protein level, we used the STRING database to construct a PPI network, which consisted of 61 nodes and 106 edges **(** Figure 4B **)**. According to the PPI network, we gained a total of 15 Hub genes **(** Figure 4C **)**. The top three hub genes were IL-1β, CCL2, and CXCR2.

### Screening characteristic genes by random forest

**FIGURE 5:**
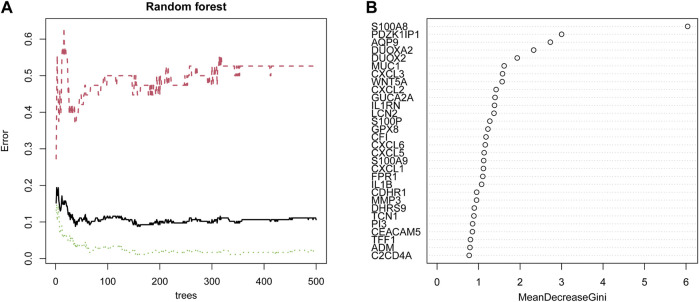
Identification of candidate genes by random forest. **(A)** The influence of the number of decision trees on the error rate. The *x*-axis represents the number of decision trees, and the *y*-axis indicates the error rate. **(B)** The importance of the top 30 genes identified by random forest. The candidate genes were identified based on the algorithm requirements of the random forest.

First, 102 DEGs were identified and incorporated into the random forest classifier. Then, we calculated the average error rate of the CD and healthy groups, respectively and observed that the error of cross validation was minimum when the number of trees was 38 (Figure 5A). Subsequently, the 30 DEGs were screened by random forest for subsequent analysis and the importance of 30 DEGs is presented in Figure 5B.

### Construction and validation of the diagnostic model

**FIGURE 6:**
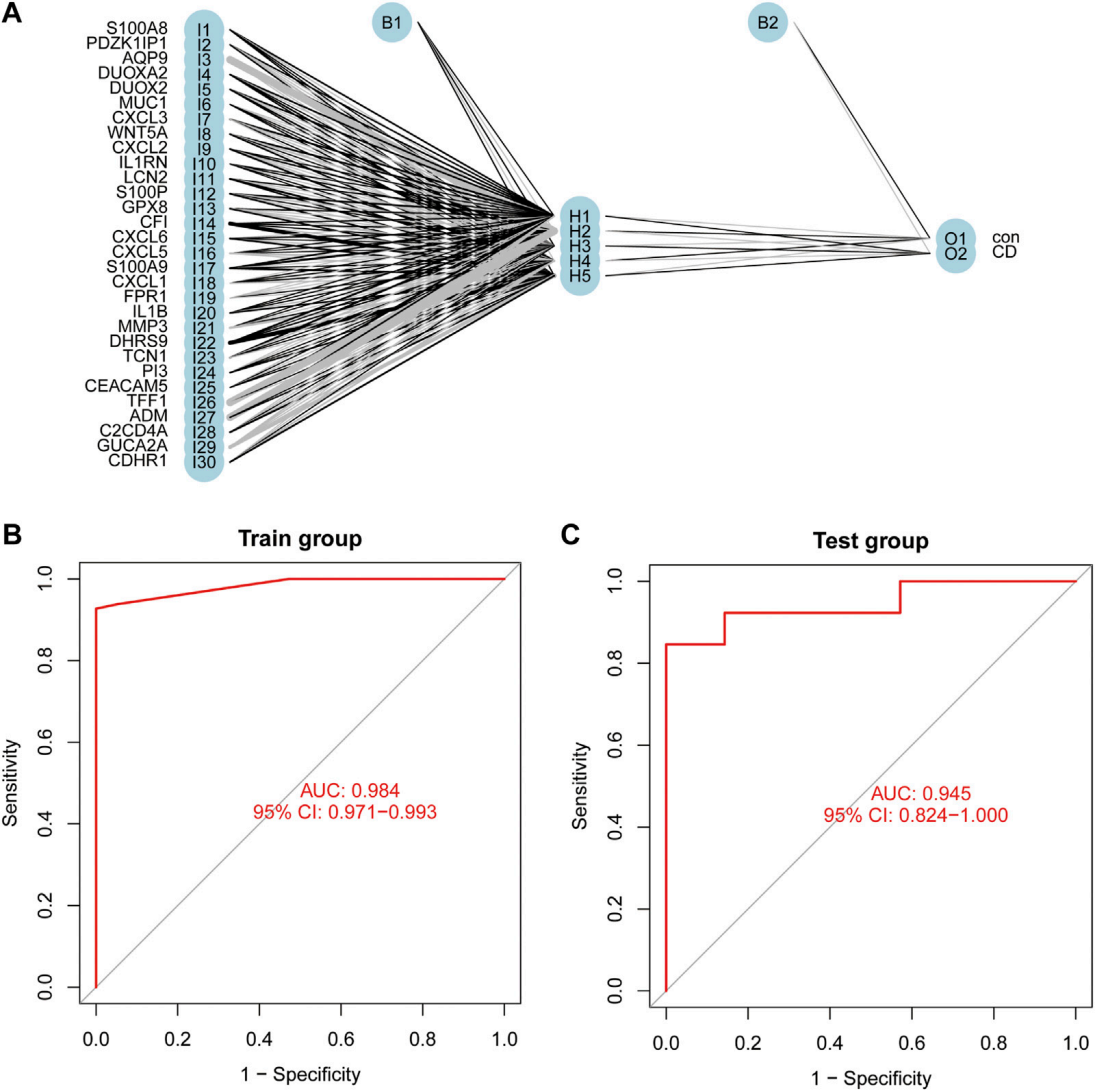
Construction of an Artificial Neural Network (ANN). **(A)** The result of ANN. **(B)** The AUC of the training cohort. **(C)** The AUC of the validation cohort.

The thirty characteristic gene scores were incorporated into the Neural Network to build a diagnostic prediction model, which included three parts: an input layer, hidden layer, and output layer (Figure 6A). A deep machine learning algorithm was performed based on the candidate gene weight to establish the ANN model, whose formula was calculated as follows: Neural CD = ∑ (Gene Score*Gene Weight). Based on the output results obtained from the neural network model, we observed that the entire training process was repeatedly conducted 2709 times. A detailed description of gene weight is shown in Table 2. Binary logistic regression analysis showed that the effect of disease location on the diagnosis of CD was not statistically significant (Table 3). The accuracy of the Neural Network to predict CD in the training and validation sets is presented in Table 4 and Table 5, respectively. We obtained positive predictive values of the training and validation sets (93.3 and 92.3, respectively), based on the Bayes algorithm. As shown in Figure 6B, the AUC of the predictive model was 0.984 [95% confidence interval (CI): 0.971–0.993], indicating that the model manifested an excellent predictive performance for CD. We used the random forest classifier to identify 30 DEGs in the validation set, which were the same as those in the training set. The AUC of the validation set was 0.945, demonstrating the credibility and stability of our model (Figure 6C).

**TABLE 2:** The gene weight of candidate genes.

Gene symbol	Gene weight
S100A8	23.07970106
PDZK1IP1	17.22093684
AQP9	23.43978408
DUOXA2	24.11507018
DUOX2	23.74934357
MUC1	−0.79382263
CXCL3	31.40582089
WNT5A	31.06980029
CXCL2	1.26885933
IL1RN	2.39195034
LCN2	7.68460207
S100P	32.09364659
GPX8	13.60179552
CFI	13.42181831
CXCL6	−1.68997642
CXCL5	4.59815424
S100A9	24.38535677
CXCL1	−1.02826724
FPR1	−2.32435006
IL1B	5.20656088
MMP3	0.1723395
DHRS9	35.72181487
TCN1	33.86358796
PI3	−0.52618612
CEACAM5	−3.4219667
TFF1	34.0373206
ADM	17.85158596
C2CD4A	−0.44643442
GUCA2A	−35.67123135
CDHR1	−1.0287512

**TABLE 3:** Binary logistic regression analysis.

		B	S.E.	Wald	df	Sig.	Exp(B)	95% C.I. for EXP(B)	
								Lower	Upper
Step 1^a^	Disease location	−.985	.533	3.409	1	.065	.373	.131	1.062
	Gene score	4.290	.901	22.654	1	.000	72.946	12.469	426.739
	Constant	−1.169	.877	1.776	1	.183	.311		

a Variable(s) entered on step 1: Disease location, Gene score.

**TABLE 4:** The accuracy of this model for predicting Crohn’s disease in the training cohort.

	Normal	Crohn’s disease	Total
Normal	37	1	38
Crohn’s disease	12	166	178

**TABLE 5:** The accuracy of this model for predicting Crohn’s disease in the validation cohort.

	Normal	Crohn’s disease	Total
Normal	3	4	7
Crohn’s disease	1	12	13

### Immune infiltration analysis

**FIGURE 7:**
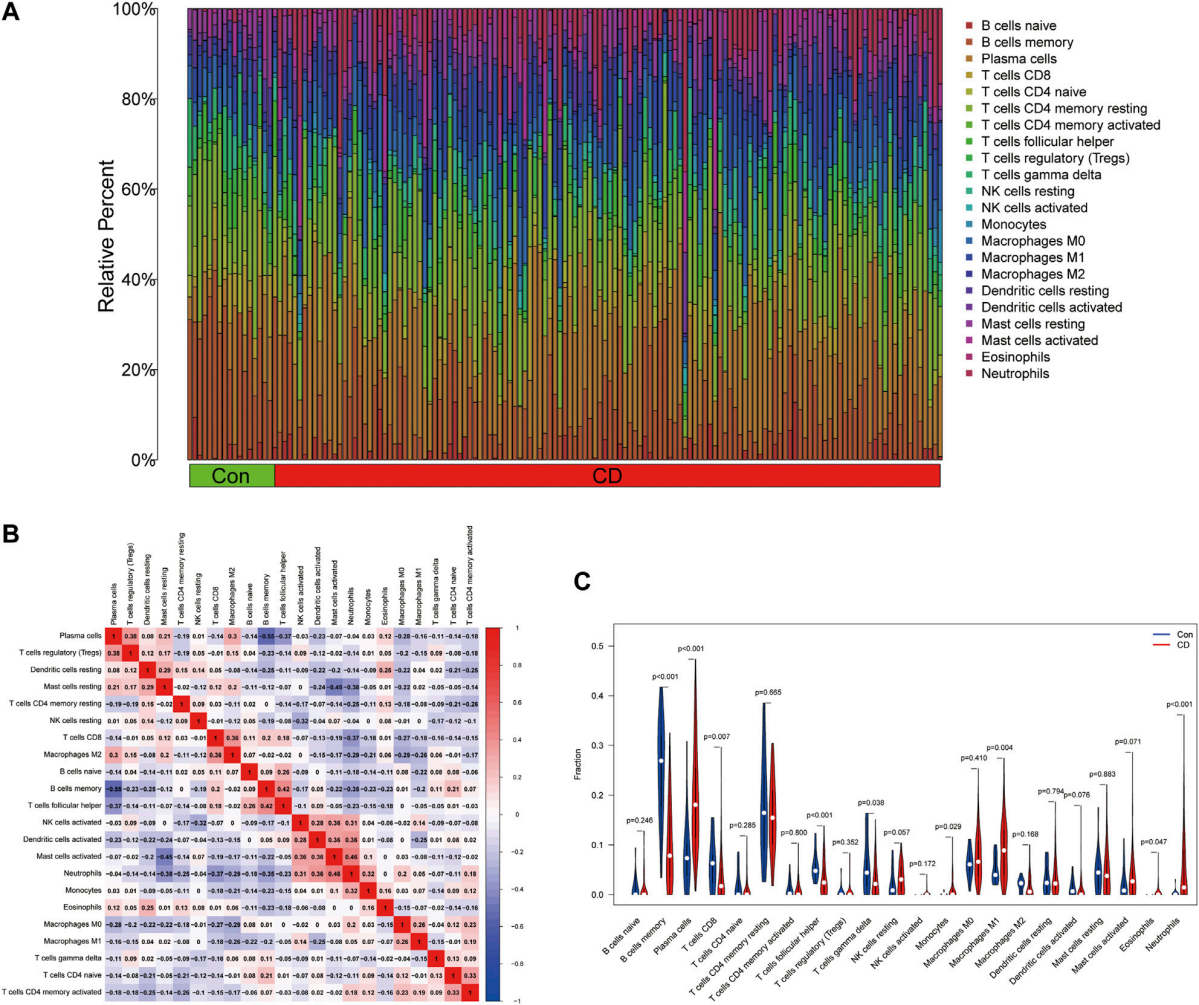
Estimation of the immune composition in tissues using CIBERSORT. **(A)** The proportion of 22 types of immune cells in CD patients and healthy individuals. **(B)** Correlation heatmap of 22 types of immune cells. **(C)** Differences in the amount of immune cell infiltration between CD and control samples.

To evaluate the proportion of the 22 types of immune cells in the CD and normal groups, we used the CIBERSORT algorithm and the results are shown as a bar plot (Figure 7A). The relevant heatmap analysis of immune cells is shown in Figure 7B, in which neutrophils were positively related to activated mast cells (*r* = 0.46), whereas memory B cells were negatively correlated with plasma cells (*r* = −0.55). Violin plots (Figure 7C) showing the immune landscape derived from the CIBERSORT algorithm revealed that CD patients had higher levels of plasma cells, CD8 T cells, follicular helper T cells, monocytes, M1 macrophages, eosinophils, and neutrophils compared with the normal group (*p* < 0.05). Consequently, these findings indicated a considerable difference in immune cell composition between the CD and healthy groups.

## Discussion

CD is a global public health problem, which threatens human health and living quality. Although the etiology of CD remains unclear, accumulating evidence indicates that the initiation of CD is significantly correlated with immunological lesions and the prominent infiltration of immune cells including T lymphocytes, macrophages, neutrophils, and plasma cells (Ramos and Papadakis, 2019). Despite its low mortality rate, its high disability rate and expense require systematic healthcare management (Hsieh et al., 2020). The first step for the effective healthcare management of CD is a definitive diagnosis, which is still a big challenge for physicians. Therefore, the development of an effective tool to diagnose CD is urgently required. A predictive model was developed to diagnose CD on the basis of deep machine learning algorithms, which has already been widely applied to the medical field to solve complicated clinical problems.

In the present study, we identified 102 DEGs associated with CD from two publicly available datasets and obtained candidate genes by a random forest classifier. GO and KEGG enrichment analysis of DEGs suggested a link between inflammatory responses and the occurrence of CD, which was reported previously (Banks et al., 2003). Singh et al. found a marked increase in cytokines and chemokines in CD patients compared with control donors, which aggravated intestinal inflammation and injury (Singh et al., 2016). Cytokines mediate inflammation, and pro-inflammatory cytokines and anti-inflammatory cytokines have a crucial role in regulating intestinal homeostasis (Moldoveanu et al., 2015; Chen et al., 2021). The aim of the targeted treatment of CD is to balance immune responses by modulating pro-inflammation and intensifying anti-inflammation. Furthermore, the top three hub genes in the PPI network were IL-1β, CCL2, and CXCR2. CCL2 and CXCR2, which belong to different subfamilies of chemokines, functioned as active biomarkers (Mello et al., 2021). Li et al. reported that CXCR2, a specific receptor for CXCL2/CXCL5, which are overexpressed in mesenchymal stromal cells (MSCs), promoted the migration of MSCs to sites of damage and achieved good therapeutic effects in an IBD mouse model (Li et al., 2021). Of note, the hub genes based on the PPI network might have an indispensable role in the pathogenesis of CD. Previous investigations indicated that IL-1β acted as an inflammasome and was overexpressed in the intestinal mucosa of CD patients (Moriyama et al., 2005; Mao et al., 2018). Inflammasomes secreted by immune cells have a pivotal role in the perpetuation of chronic and active inflammation (Zhen and Zhang, 2019; Jackson and Theiss, 2020; Mahapatro et al., 2021).

In addition, CIBERSORT analysis presented a landscape of 22 immune cells and we analyzed the differences between CD patients and healthy donors. Innate immunity is an immediate non-specific response, in which pathogens are recognized and innate immune cells include macrophages, neutrophils, dendritic cells, and natural killer cells are activated (Xu et al., 2014; Holleran et al., 2017). The intestinal innate immune system protects the mucosa by immune defense and surveillance. Recent research demonstrated that the innate immune response is equally significant as adaptive immunity in CD. In addition to defense against many aggressors, innate immunity also contributes to abnormal immune responses, such as autophagy, innate microbial sensing, and antimicrobial peptide production, which are linked to CD pathogenesis (Geremia et al., 2014; Zhou et al., 2019; Castellanos and Longman, 2020). Based on our findings, the numbers of plasma cells and CD8 T cells in patients with CD were higher than in the normal group, as were the follicular helper T cells, monocytes, M1 macrophages, and neutrophils.

To the best of our knowledge, this is the first study to combine a random forest classifier with an ANN algorithm to develop a diagnostic prediction model for CD, which had a good performance when discriminating CD from healthy controls. S100A8 was the best candidate gene identified by a random forest classifier in our study. A recent study reported that the expression levels of serum biomarkers, including S100A8, were elevated in patients who later had a disease relapse (Kessel et al., 2021). It was also reported that maintaining the production balance of S100A8 might be involved in intestinal homeostasis (Fujita et al., 2018; Loh et al., 2021). Due to its high efficiency and accuracy, random forest algorithms as a type of ensemble learning method have been successful in the prediction and identification of clinical disease (Savargiv et al., 2021). Wang et al. developed a model to predict coronary artery disease using a random forest algorithm, which had good discrimination abilities (Wang et al., 2021). Another study demonstrated that endobronchial optical coherence tomography based on a random forest algorithm was effective for the detection of early malignant pulmonary disease (Ding et al., 2021). A recent study reported that Support Vector Machines and multi-layer neural network models were developed to predict a protein’s post translational modification sites, which contributed to solving a difficult problem in the field of molecular biology (Bao et al., 2018). ANN have also increasingly been used in gastrointestinal disease because of its outstanding performance in diagnostic and prognostic prediction (Cao et al., 2021). In the 1990s, Karakitsos et al. reported the use of ANN to detect malignant gastric lesions with a discrimination performance of 97% (Karakitsos et al., 1998). Furthermore, a previous study reported combining machine algorithms to predict the migration of cancer cells (Zhang et al., 2018). Moreover, a joint machine algorithm of a random forest and ANN contributed to a diagnosis of non-tumor diseases including heart failure (Tian et al., 2020). Li et al. also successfully developed a novel diagnostic model for UC integrating a random forest and ANN algorithm (Li et al., 2020). Therefore, corresponding deep machine learning should be applicable for the diagnosis of CD. Several studies have focused on potential biomarkers as predictive factors to develop a model for CD diagnosis. The prediction performance in our study had better accuracy and precision compared with previous investigations. It is essential to further explore the use of joint algorithms for the prognosis and treatment of inflammatory bowel disease in the future.

Although this study developed a new prediction model for CD based on high-throughput sequencing data, there were several limitations that should be taken into account. First, our study was a retrospective analysis and the sample size was relatively small. Due to the use of a public limited database, there was a mismatch between the CD patients and controls. In the future, a prospective study with a large sample of matching data is required to demonstrate the reliability of this finding. Second, our training cohort consisted of different platform datasets; therefore, the these differences in study design and measurement errors might have influenced the study results. Third, due to the lack of laboratory investigation, the specific role of each immune cell type in the pathogenesis of CD was not be completely elucidated.

However, this research provides a novel insight into the accurate diagnosis of CD, and identified potential biomarkers for precise treatment.

## Conclusion

Based on the points mentioned above, the correlation analysis of immune cells helped us to enhance our current understanding of the etiology of CD. A co-operative diagnosis model using a random forest and ANN was an effective diagnostic method for CD, and had good predictive performance in the training and validation sets. However, clinical trials are needed to investigate and validate the credibility of our findings.

## Data Availability

The original contributions presented in the study are included in the article/Supplementary material, and further inquiries can be directed to the corresponding author.
